# Supplementary material for: Wastewater-Based Surveillance Reveals the Effectiveness of the First COVID-19 Vaccination Campaigns in Assisted Living Facilities
Source: Int J Environ Res Public Health. 2024 Sep 23;21(9):1259. doi: 10.3390/ijerph21091259 (PMC11431242; doi:10.3390/ijerph21091259)
Supplement: Supplementary file 1 [file ijerph-21-01259-s001.zip › ijerph-3165586-supplementary.pdf]

Supplementary Materials

Table S1. Facility 1 Wastewater Surveillance Data

Sample Collection Date	SARS-CoV-2 Results Quantitative (Log10 copies/L)
1/7/21	7.13
1/10/21	5.06
1/12/21	5.87
1/14/21	3.99
1/17/21	4.26
1/19/21	5.07
1/21/21	ND
1/24/21	3.45
1/26/21	ND
1/28/21	4.59
1/31/21	4.65
2/2/21	3.92
2/4/21	5.16
2/7/21	3.89
2/9/21	4.46
2/12/21	ND
2/16/21	ND
2/18/21	ND
2/23/21	ND
2/28/21	ND
3/2/21	ND
3/4/21	ND
3/7/21	ND
3/9/21	ND
3/11/21	ND
3/14/21	ND
3/18/21	ND
3/21/21	ND
3/22/21	ND
3/25/21	ND
3/28/21	ND
3/29/21	ND
4/4/21	ND

4/6/21	ND
4/8/21	ND
4/11/21	ND
4/13/21	ND
4/18/21	ND
4/20/21	ND
4/25/21	4.25
4/27/21	ND

Table S2. Facility 2 Wastewater Surveillance Data

Sample Collection Start Date	SARS-CoV-2 Results Quantitative (Log10 copies/L)
1/5/21	ND
1/7/21	5.67
1/10/21	ND
1/12/21	ND
1/14/21	ND
1/17/21	ND
1/19/21	ND
1/21/21	3.81
1/24/21	4.24
1/26/21	ND
1/28/21	ND
1/31/21	ND
2/2/21	3.84
2/4/21	ND
2/7/21	ND
2/9/21	ND
2/11/21	ND
2/16/21	3.25
2/18/21	ND
2/23/21	ND
2/28/21	ND
3/2/21	ND
3/7/21	ND
3/9/21	ND
3/11/21	ND
3/14/21	ND
3/16/21	ND
3/18/21	ND
3/21/21	ND
3/22/21	ND
3/25/21	ND
3/28/21	3.43
3/29/21	ND
4/4/21	ND
4/6/21	ND
4/8/21	ND
4/11/21	ND

4/13/21	ND
4/18/21	ND
4/20/21	ND
4/25/21	ND
4/27/21	ND

Table S3. Facility 3 Wastewater Surveillance Data

Sample Collection Start Date	SARS-CoV-2 Results Quantitative (Log10 copies/L)
1/5/21	ND
1/7/21	ND
1/10/21	ND
1/12/21	4.78
1/14/21	ND
1/17/21	ND
1/19/21	3.76
1/21/21	ND
1/24/21	ND
1/26/21	ND
1/28/21	ND
1/31/21	ND
2/2/21	4.42
2/4/21	ND
2/7/21	ND
2/9/21	ND
2/11/21	ND
2/16/21	ND
2/18/21	ND
2/23/21	ND
2/28/21	ND
3/2/21	ND
3/7/21	ND
3/9/21	ND
3/11/21	ND
3/14/21	3.55
3/16/21	ND
3/18/21	ND
3/21/21	ND
3/22/21	ND
3/25/21	ND
3/28/21	ND
3/29/21	ND
4/4/21	3.84
4/6/21	ND
4/8/21	ND
4/11/21	ND

4/13/21	ND
4/18/21	ND
4/20/21	ND
4/25/21	ND
4/27/21	3.78

Table S4. Facility 4 Wastewater Surveillance Data

Sample Collection Start Date	SARS-CoV-2 Results Quantitative (Log10 copies/L)
1/5/21	ND
1/7/21	4.26
1/10/21	ND
1/12/21	ND
1/14/21	ND
1/17/21	ND
1/19/21	3.77
1/21/21	3.97
1/24/21	3.93
1/26/21	ND
1/28/21	4.69
1/31/21	ND
2/2/21	ND
2/4/21	ND
2/7/21	ND
2/9/21	ND
2/11/21	ND
2/16/21	ND
2/18/21	3.52
2/23/21	4.04
2/28/21	ND
3/2/21	ND
3/7/21	ND
3/9/21	ND
3/11/21	ND
3/14/21	ND
3/16/21	ND
3/18/21	ND
3/21/21	ND
3/22/21	ND
3/25/21	ND
3/28/21	ND
3/29/21	ND
4/4/21	ND
4/6/21	3.65
4/8/21	ND
4/11/21	ND

4/13/21	ND
4/18/21	ND
4/20/21	ND
4/25/21	ND
4/27/21	6.02
